# Transpiration Reduction in Maize (*Zea mays* L) in Response to Soil Drying

**DOI:** 10.3389/fpls.2019.01695

**Published:** 2020-01-23

**Authors:** Faisal Hayat, Mutez Ali Ahmed, Mohsen Zarebanadkouki, Mathieu Javaux, Gaochao Cai, Andrea Carminati

**Affiliations:** ^1^Chair of Soil Physics, University of Bayreuth, Bayreuth, Germany; ^2^Division of Soil Hydrology, University of Göttingen, Göttingen, Germany; ^3^Earth and Life Institute-Environmental Sciences, Universite Catholique de Louvain, Louvain la Neuve, Belgium; ^4^Agrosphere (IBG-3), Forschungszentrum Juelich GmbH, Juelich, Germany

**Keywords:** maize (*Zea mays* L), pressure chamber, soil drying, stomatal closure, transpiration rates

## Abstract

The relationship between leaf water potential, soil water potential, and transpiration depends on soil and plant hydraulics and stomata regulation. Recent concepts of stomatal response to soil drying relate stomatal regulation to plant hydraulics, neglecting the loss of soil hydraulic conductance around the roots. Our objective was to measure the effect of soil drying on the soil-plant hydraulic conductance of maize and to test whether stomatal regulation avoids a loss of soil-plant hydraulic conductance in drying soils. We combined a root pressure chamber, in which the soil-root system is pressurized to maintain the leaf xylem at atmospheric pressure, with sap flow sensors to measure transpiration rate. The method provides accurate and high temporal resolution measurements of the relationship between transpiration rate and xylem leaf water potential. A simple soil-plant hydraulic model describing the flow of water across the soil, root, and xylem was used to simulate the relationship between leaf water potential and transpiration rate. The experiments were carried out with 5-week-old maize grown in cylinders of 9 cm diameter and 30 cm height filled with silty soil. The measurements were performed at four different soil water contents (WC). The results showed that the relationship between transpiration and leaf water potential was linear in wet soils, but as the soil dried, the xylem tension increased, and nonlinearities were observed at high transpiration rates. Nonlinearity in the relationship between transpiration and leaf water potential indicated a decrease in the soil-plant hydraulic conductance, which was explained by the loss of hydraulic conductivity around the roots. The hydraulic model well reproduced the observed leaf water potential. Parallel experiments performed with plants not being pressurized showed that plants closed stomata when the soil-plant hydraulic conductance decreased, maintaining the linearity between leaf water potential and transpiration rate. We conclude that stomata closure during soil drying is caused by the loss of soil hydraulic conductivity in a predictable way.

## Introduction

Drought is a primary constraint to plant growth and crop production worldwide. Mechanisms by which drought impacts plant growth are complex and involve feedbacks between stomata regulation, plant hydraulics and soil drying. A hydraulic framework is helpful to understand the physical constraints to transpiration (Sperry and Love, 2015). The soil-plant atmospheric continuum is described as a network of elements connected in series and in parallel (Cowan, 1965; Sperry et al., 1998; Draye et al., 2010; Mencuccini et al., 2019). Each element is characterized by hydraulic conductances (which can be variable) and capacitances. Water flows from soil to the roots, and then along the xylem till the leaf tissues and stomata, where it evaporates into the atmosphere following the cohesion-tension theory (Pickard, 1981; Sperry et al., 1998). The driving force for transpiration is the water tension generated in the leaves because of the evaporating water. The tension propagates down along the xylem to the roots and to the soil. The hydraulic conductivities of the xylem, of the roots and of the soil are extremely variable. Xylem vessels tend to cavitate at high tension, causing a large drop in the axial conductance of the xylem (Sperry et al., 1998). The radial conductance of the root is also variable and it is affected by anatomical changes as well as by the expression of aquaporin (Ehlert et al., 2009; Redondo et al., 2009; Simonneau et al., 2009; Knipfer et al., 2011; Chaumont and Tyerman, 2014). Finally, the soil hydraulic conductivity determines the ease of water flow through the soil. Its conductivity decreases by several orders of magnitude as the soil dries, and it might become smaller than that of roots (Gardner, 1960; Draye et al., 2010). Eventually, when plants are exposed to severe drying, their roots shrink and lose part of their contact to the soil (Carminati et al., 2013), which further decreases the conductance between rhizosphere and root. On the other hand, plants can close this gap and attenuate the drop in conductivity by secreting mucilage (Carminati et al., 2010) or by growing root hairs (Carminati et al., 2017).

Soil drying triggers a gradual closure of stomata and a reduction in transpiration rate (Carter et al., 1980; Meyer and Green, 1980; Bates et al., 1981; Comstock, 2002; Sinclair et al., 2005). Stomatal closure depends on both hydraulic and hormonal signals, such as abscisic acid (ABA) (Tardieu and Davies, 1993; Brodribb and McAdam, 2017 and Buckley, 2017). Independently from the mechanism by which stomata close, it has been proposed that stomatal regulation avoids excessive drop in leaf water potential by responding to nonlinearities in the relationship between transpiration rate and leaf water potential (Sperry and Love, 2015; Sperry et al., 2016). However, there is limited experimental evidence that stomatal regulation prevents and responds to drop in soil-plant hydraulic conductance. Additionally, most of the studies linking stomatal regulation to plant hydraulics focus on xylem vulnerability as the primary constraint on water flow in soil and plants (Anderegg et al., 2017), neglecting the explicit role of soil hydraulic conductivity.

Our objective was to test whether stomata close when the soil-plant hydraulic conductance drops during soil drying. Here, we use a soil-plant hydraulic model that solves the radial flow of water around a representative single root (Gardner, 1960; Van Lier et al., 2008) and water flow in the plant (Sperry et al., 1998) to test whether the drop in hydraulic conductance can be predicted based on the loss of soil hydraulic conductance.

Experimentally, we applied the pressure chamber method (Passioura, 1980) to maize (*Zea mays* L) growing in silty soil. The root-soil system of intact transpiring plants is pressurized to maintain the leaf xylem at atmospheric pressure. The applied pressure is then equivalent to the tension of water in the leaf xylem (Passioura, 1980). The method allows accurate measurements at high temporal resolution of leaf water potential for varying transpiration rates and soil water potential. Furthermore, we measured transpiration rates for pressurized (in the pressure chamber) and not-pressurized (outside the pressure chamber) plants to test to what extent leaf tension controls stomata closure in drying soils.

## Materials and Methods

### Soil and Plant Preparation

Three replicates of maize (*Zea mays* L.) were grown in PVC pots with 30 cm of height and 9 cm of diameter. The pots were filled with a mixture of silt and quartz sand (1:1 ratio) {{mdash}} which were sieved to a particle diameter < 1 mm. The soil was poured into each pot to achieve a bulk density of 1.4 g cm^-3^. The soil surface of each pot was covered with fine gravels (2–3.5 mm) to minimize evaporation from the soil surface. Several holes with a diameter of 1.5 mm were drilled at the bottom and sides of the pots to allow, respectively, water drainage and lateral injection of water using a fine needle. Five holes were placed with diameter of 5 mm and with a distance of 5 cm from each other at the sides of the pots to measure soil water content using a TDR (time domain reflectometer, FOM/mts, E-Test (IA PAS), Lublin, Poland). The soil hydraulic properties were estimated using extended evaporation method (Peters and Durner, 2008; Schindler et al., 2010). The implementation of this method using Hyprop (Meters, Munich, Germany) and the parameterization of retention curve and soil hydraulic conductivity has been described in Hayat et al (2018).

Maize seeds were germinated on moist filter paper for 48 h and the seedlings were planted in the containers. The plants were grown for 40 days in a climate room with a photoperiod of 14 h, day/night temperature of 25°C/22°C, relative humidity of 60% and light intensity 200 µmol m^-2^ s^-1^. During the first three weeks, the plants were irrigated every third day by immersing the pots in a nutrient solution to achieve an average soil water content of 25%. Afterward, the soil water contents were adjusted to the following scenarios: i) water content of 21%–25% (wet soil); ii) water content of 12%–13% (midwet soil); iii) water content of 9%–10% (middry soil); iv) and water content of 6%–6.5% (dry soil). The soil water contents were measured every third day using TDR. The soil moisture content was measured at five different heights (5, 10, 15, 20, 25, and 30 cm).

### Transpiration Measurements

Prior to the experiment, we measured soil water contents at five different heights as described above. Afterwards, transpiration rates for each scenario were recorded by Sap Flow Sensors SGA9 (Dynamax Inc, USA). This nonintrusive, energy balance sensor measures the amount of heat carried by the sap and converts into real-time transpiration rate.

Transpiration rates were also measured by weighing the plants before and after the recordings, and the decrease in weight was compared to the cumulative flow measured with the sap flow sensors (**Figure S1A**). A LED lamp (GC 9, photo flux density (15 cm), 2450 µmol m^-2^ s^-1^, Greenception GmbH, Hamburg) was installed at a distance of 16 cm above the shoots (**Figure S1B**). Transpiration was increased in four steps (from low to high transpiration) by increasing photosynthetic photon intensity. Transpiration was measured for a period of one and a half hour for each step. At the end of transpiration measurements, water was injected in the pot through the holes to bring the soil to the initial soil water content.

### Pressure Chamber

Xylem water potential of transpiring plants was measured using the pressure chamber method, based on Passioura (1980). We started the experiment when plants were 40 days old. Briefly, the soil core and the roots were put inside the pressure chamber in such a way that the shoot remained outside and it was carefully sealed to avoid air leakage (**Figure S1B**). One leaf was cut and the pressure in the chamber was increased (using 99.9% vol. N_2_) until a water droplet appeared on a cut leaf (**Figure S1C**). The pressure needed to keep a drop of water at the cut end of the leaves is numerically equal to the tension in the xylem (Passioura, 1980). Transpiration was increased stepwise by imposing leaves to four increasing photosynthetic photon intensities. In each step, we let the plant to transpire for 1.5 h. During this time, transpiration was measured using a sap flow sensor that was installed on the stem of the plant. The measurements were performed for four scenarios of moisture levels and four transpiration rates. To reveal the effect of soil and plant pressurizing on the transpiration rate (stomata closure), each measurement was performed with and without pressurizing the soil.

### Soil-Plant Hydraulic Model

We used a simple model to estimate the water flow in the soil-plant continuum. The model was represented as a series of hydraulic resistances (and one capacitance in the soil) between the bulk soil and the leaves. The flux of water in the soil *q* [cm s^-1^] is calculated using a cylindrical model as a function of radial distance *r* to the root center:

(1) $$q\left(r\right)=-k_{soil}\left(\psi \right)\frac{\partial \psi }{\partial r}$$

where *k_soil_* is the soil conductivity [cm s^-1^] (when the matric potential is expressed as hydraulic head, i.e., 1 hPa ≈ 1 cm), which is function of matric potential *ψ* [hPa], and $\frac{\partial \psi }{\partial r}$ is the gradient in matric potential. As boundary condition at the root-soil interface, we set $q\left(r_{0}\right)=-\frac{T}{2\pi r_{0}L}$ where *T* is the transpiration rate [cm^3^ s^-1^], *r*_0_ is the root radius [cm] and *L* is the active root length [cm]. We imposed no flow at the outer root radius *r_b_* [cm], i.e., *q*(*r_b_*) = 0, where $r_{b}=\sqrt{\frac{V}{\pi L}}\;$here *V* is the soil volume [cm^3^] and *ψ* = *ψ_b_*.

The soil hydraulic conductivity *k_soil_* [cm s^-1^] is parameterized using Brooks and Corey model (Brooks and Corey, 1964):

(2) $$k_{soil}\left(\psi \right)=\;k_{sat}{\left(\frac{\psi }{\psi_{o}}\right)}^{\tau }$$

where *k_sat_* is the soil saturated hydraulic conductivity [cm s^-1^], *τ* is a fitting parameter [-], *ψ_o_* is the soil air entry value [hPa^-1^].

Equation (1) is linearized following (van Lier et al., 2006; Schröder et al., 2007), who assumed a steady-rate behavior and used the matric flux potential [cm^2^ s^-1^]:

(3) $$\text{Φ}\left(\psi \right)=\;\int_{-\infty }^{\psi }k\left(x\right)dx$$

Following this approach, we obtain:

(4) $${\text{Φ}}_{r,s}=-\frac{T}{2\pi r_{0}L}\left(\frac{r_{0}}{2}-r_{0}r_{b}^{2}\frac{ln\left(r_{b}/r_{0}\right)}{r_{b}^{2}-\;r_{0}^{2}}\right)+\;{\text{Φ}}_{b}$$

where Φ*_b_* is obtained from inserting *ψ_b_* in Eq. (2–3). Inverting Eq. 3 and using the parameterization of Eq. 2, from Φ*_r,s_* (Eq. 4) we obtain *ψ_r,s_*.

Knowing the transpiration rate and the plant hydraulic conductance, *K_plant_* [cm^3^ hPa^-1^ s^-1^], the dissipation of water potential within the plant is calculated as:

(5) $$T=\;K_{plant}\left(\psi_{leaf,x}-\;\psi_{r,s}\right)$$

where *ψ_leaf_*,*_x_* is the water potential in the leaf xylem [hPa].

In this model, we assumed that: 1) the total length of the roots taking up water is *L*; 2) all the roots take up water at similar rate; 3) the soil water potential is at at distance *r_b_* from the root center is equal for all roots; 4) there is no cavitation in the xylem. The last assumption is justified by the fact that during the measurements the plant was maintained pressurized and water in the leaf xylem was at atmospheric pressure. The illustration of these parameters is shown in **Figure S2**.

The model allows to calculate the leaf water potential *ψ_leaf_* for varying soil water potential *ψ_b_* and transpiration rates T. The model requires the parameters *K_plant_*, *L*, *r_b_*, *r*_0_ and the function *k_soil_* (*ψ*) (Eq. 2). *k_soil_* (*ψ*) was measured and parameterized independently (**Figure S3**). The root radius *r*_0_ was set to 0.05 cm. *r_b_* is calculated as $r_{b}=\sqrt{\frac{V}{\pi L}}$. The independent parameters were *K_plant_* and *L* and were adjusted to best reproduce the measured balancing pressure *P* [hPa] for the different transpiration rates and soil water potentials.

The root pressure chamber is numerically equal to minus of the leaf water potential:

(6) $$P\;=\;-{\text{ψ}}_{leaf,x}$$

assuming that gradients in osmotic potential are negligible. Additionally, the root length was independently measured using WinRhizo and then compared to the fitted *L*.

## Statistical Analysis

The effects of soil water content, light intensity, pressurization, and the interactions between them on transpiration were analysed using *N*-way analysis of variance (ANOVA) followed by Tukey-Kramer multiple comparison tests. In all cases, *p* < 0.05 was taken as the lowest level of significance. Matlab (**9.5.0**) and the corresponding statistic packages were used to perform all the statistical analysis.

## Results

The soil water retention and unsaturated conductivity curves obtained by fitting the evaporation method are shown in **Figure S3A**. The fitting parameters of the water retention curve were further used to estimate the soil hydraulic conductivity using Brooks and Corey parameterization (Brooks and Corey, 1964) (**Figure S3B**).

**Figure 1:**
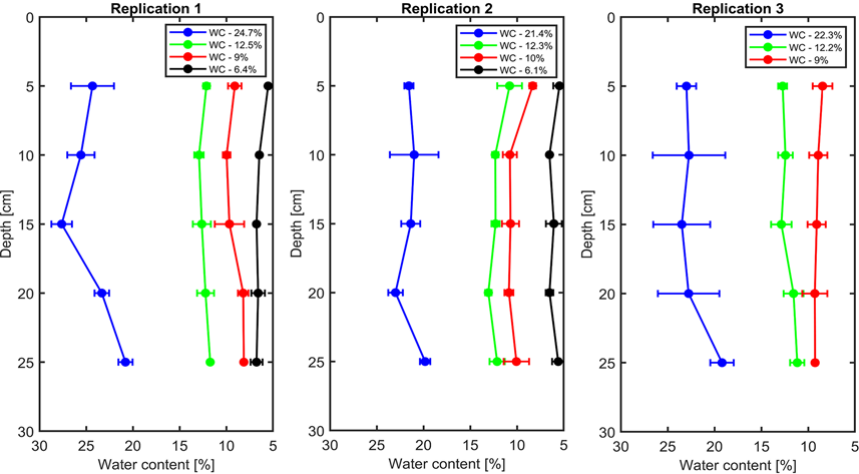
Vertical profiles of volumetric soil water content in each replication.

The soil water content profiles were measured by the TDR in all replications that are shown in **Figure 1**. The measurements showed that the distribution of water content was relatively homogeneous throughout the soil profile.

We calibrated the sap flow sensors using the gravimetric measurements (**Figure S4**). The transpiration rate measured by sap flow was linearly related to the gravimetric measurements. We repeated the calibration for each measurement (e.g., for each water content and for each sample).

**Figure 2:**
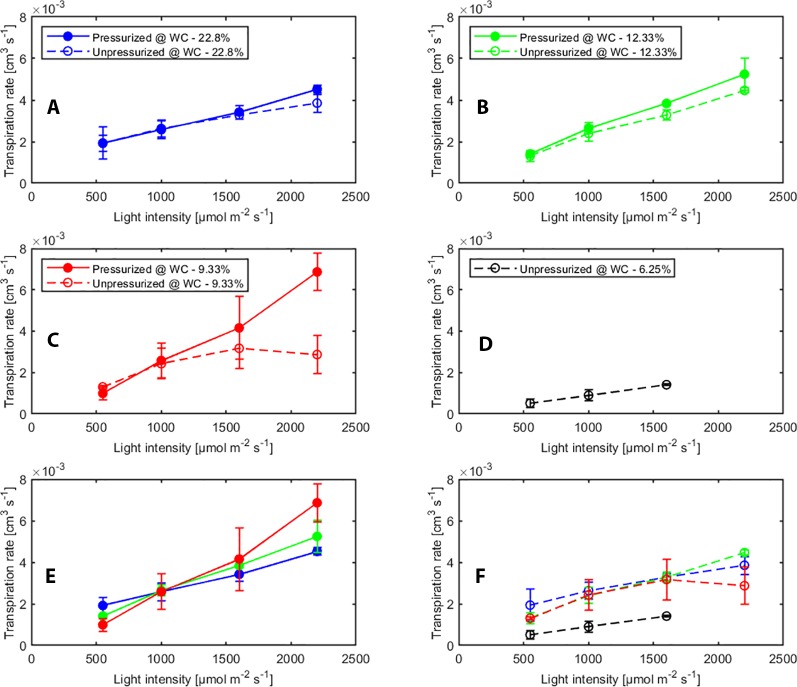
Effect of light intensity and pressurization on transpiration rates for varying soil water contents. **(A**–**D)** Effect of pressurization on transpiration. **(E)** Effect of light intensity and soil moisture on transpiration in pressurized and **(F)** unpressurized plants.

The effect of pressurization and light intensity on averaged transpiration rates (measured with sap flow sensors) with and without pressurization at each water content are shown in **Figure 2**. In general, we observed a slightly higher transpiration rate when the plants were pressurized. This indicates that when plants were pressurized and water in the leaf xylem was at atmospheric pressure, the stomata were more open. However, as long as the soil was wet or the light intensity was low, transpiration rate increased with increasing light intensity under both, pressurized and not pressurized conditions. In contrast, in dry soil (WC = 9.33%) under not pressurized conditions transpiration dropped significantly (*p* < 0.05, Tukey-Kramer test) at high photosynthetic photon intensity (at 2000 µmol m^-2^ s^-1^) (**Figure 2C**). At the tested soil moistures, pressurization prevented stomatal closure at all soil moistures. **Figure 2E** shows a linear response of transpiration to increasing light intensity and the increase in transpiration was even more marked in dry soil (**Figure 2E**).

We tested the statistical significance of the effect of different factors (i.e., pressurization, soil water content and light intensity) and the interaction on transpiration rate by ANOVA (see **Supplementary Material Table S1**). Transpiration rate was significantly influenced by light intensity and pressurization. The effect of pressurization interacted with that of light intensity on transpiration rate. This implies that for different light intensities the impact of pressurization was different. Soil water content and its interaction with other two factors did not show significant impact, which was possibly because of limited measurements at low soil moistures.

**Figure 3:**
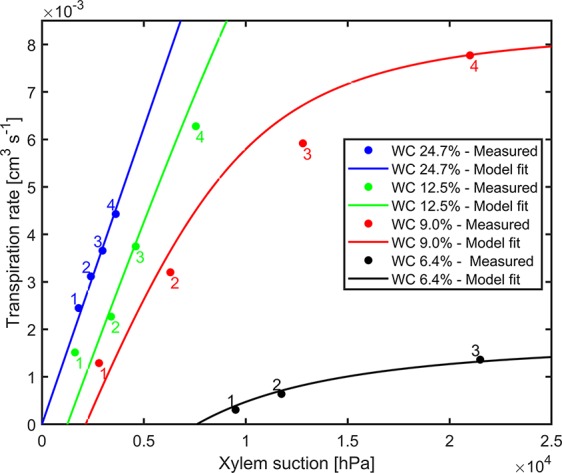
Measured xylem suction and transpiration rate for decreasing water contents (WC) and increasing light intensity (1–4) for replicate 1 (2 and 3 are shown as **Supplementary Material**). The solid lines are the model fits.

The comprehensive data sets of transpiration rates, measured xylem tension, and the model fitting for different water contents for replication 1 are shown in **Figure 3**. Dots are transpiration rates and leaf water potential measured when plants were pressurized for four imposed photosynthetic photon intensities (550, 1,000, 1,600, and 2,200 µmol m^-2^ s^-1^ marked as 1–4). The solid lines are the fitting of the model. In wet soil (WC = 24.7%), the relationship between transpiration rate and xylem tension was linear. As the soil dried (WC = 12.5%, 9% and 6.4%), this relationship became nonlinear at increasing transpiration rates. The slope of linear part of the curve at high water content (at WC = 24.7%) is interpreted as the plant conductance, *K_plant_* (i.e., soil resistance is assumed to be negligible). This conductance was used in the simulations. For high water content, the conductance *K_plant_* (at WC = 24.7%) was 1.25×10^-6^ [cm^3^ hPa^-1^ s^-1^]. The total soil-plant conductance reduced dramatically in dry soils at high transpiration rates due to the drop of soil hydraulic conductivity around the roots, which is well reproduced by the soil hydraulic model. The relation between transpiration rates, measured xylem tension, and the model fitting for different water contents for replication 2 & 3 are shown in **Supplementary Material** (**Figure S5**). Conductance of the root system, active root length used in the model, and coefficient of correlation for each replication are shown in **Table 1**.

**Table 1:** The conductance of soil-root system, active root length optimized for the model and R^2^ in each replication.

Replication	*K_plant_*	*L*	R^2^
	[cm^3^ hPa^-1^ s^-1^]	[cm]	
1	1.25×10^-6^	700	0.9808
2	1.05×10^-6^	200	0.3518
3	5.63×10^-5^	350	0.8991

**Figure 4:**
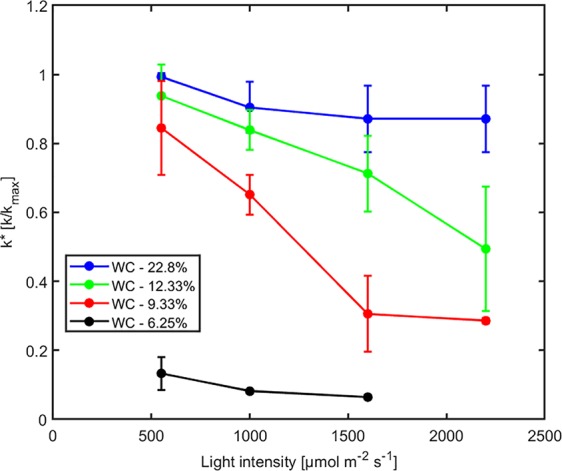
Effect of light intensity on normalized soil-plant conductance k* = k/k_max_ (where k_max_ is the soil-plant conductance in the wettest soil and lowest light intensity) at varying soil water contents (WC). Relative soil-plant conductance k* decreased with increasing light intensity due to higher transpiration rates and with decreasing soil water contents due to the decreasing soil hydraulic conductivity.

**Figure 5:**
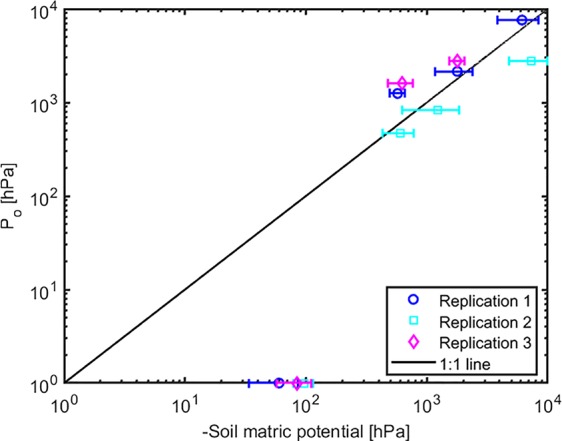
The relation between intercept (Po) and the soil matric potential. The points below (above) the 1:1 line indicate a more negative (positive) osmotic potential in the leaf xylem than in the soil.

The effect of light intensity and water content on normalized soil-plant conductance k* is shown in **Figure 4**. The k* value is the ratio of soil-plant conductance to the maximum conductance measured in wet soil and low light intensity. In general, soil water content and light intensity and their interaction affected k* extremely significantly (*p* < 0.01, **Table S2**). k* is approximately constant in wet soil at each imposed light intensity. In drier soil (WC = 12.33% and 9.33%), k* reduced with increasing light intensity. The reduction was extremely significant (*p* < 0.01, Tukey-Kramer test) at WC = 9.33% where it occurred at light intensity of ca. 1,500–2,000 µmol m^-2^ s^-1^. At WC = 12.33% the drop was only significant (*p* < 0.05, Tukey-Kramer test) at light intensity above 2,000 µmol m^-2^ s^-1^. Note that these were the conditions when transpiration was reduced in the unpressurized plants (**Figures 2B, C**). The relationship between P_0_ [hPa] (intercept of xylem pressure and transpiration rate) and minus the soil matric potential [hPa] is plotted in **Figure 5**. In principle, these values should fit unless there was a large osmotic gradient between the xylem and the soil. In dry soil, the values fitted rather well (consider that the estimation of the soil matric potential based on water retention curve are prone to errors in the dry range). In wet soil, (i.e., WC between 21.4% and 24.7%), the soil matric potential was slightly more negative than the fitted P_0_, which indicates a more positive pressure in the xylem than in the soil, possibly caused by a more negative osmotic potential in the xylem than in the soil. The difference of ca. 50 –100 hPa is not detectable at more negative soil water potential (as explained in the note above).

## Discussion and Conclusions

We measured the relationship between leaf water potential and transpiration rates in maize at various soil water contents and light intensity. From this relationship, we estimated the soil-plant hydraulic conductance and its decrease with increasing transpiration rates and decreasing soil moistures. In parallel, we have measured the transpiration rates (for unpressurized plants). We have found that reductions in transpiration occurred in correspondence to reductions in soil-plant hydraulic conductance, which were caused by the loss of soil hydraulic conductivity around roots.

Pressurization increased the transpiration rates almost at all soil water contents and each imposed light intensity (see **Figure 2**). However, this effect was particularly visible only in dry soil conditions and high light intensity. At WC = 9.33% and high light intensity (2,200 µmol m^-2^ s^-1^) pressurization increased transpiration by a factor of 3 (**Figure 2C**) compared to unpressurized plants. At this condition, the leaf potential would have been around −2.1 MPa if the plant had not been pressurized (**Figure 3**) and the relationship between leaf water potential and transpiration rate would have been extremely nonlinear (**Figure 3**, red line, point 4). At low soil water content and high light intensity the soil-plant hydraulic conductance was significantly reduced. Interestingly, the soil-plant hydraulic conductance was already reduced in wetter soil (WC = 12.33%) and at lower light intensity (WC = 9.33%, LI ≈ 1600 µmol m^-2^ s^-1^). This suggests that the drop in hydraulic conductance anticipated (and possibly triggered) the reduction in transpiration. It also shows that stomatal regulation (prevented in the pressurized plants) occurred when the soil-plant hydraulic conductance decreased.

The relationship between leaf xylem tension and transpiration rate (under pressure) was linear in wet soils and became nonlinear at drier soil conditions and increasing transpiration rates (**Figure 3**). The nonlinearity in this relationship corresponds to a decrease in soil-plant conductance shown in **Figure 4**. This finding is consistent with previous measurements with barley (*Hordeum vulgare*) (Carminati et al., 2017) and wheat (*Triticum*) (Passioura, 1980), and fits well with early model of root water uptake (Gardner and Ehlig, 1963).

The soil-root hydraulics model was capable to reproduce the measured relationship between xylem tension and transpiration rate. The only unknown parameters of the model were: 1) the plant conductance *K_plant_*, equal to the inverse of the slope of the xylem suction versus transpiration rate at high WC; and 2) the active root length *L*, which is the effective length of the roots actually taking up water, and which determines the onset of nonlinearity in the curves. The best fits were obtained with *L* = 200, 350, and 700 cm. Note that the measured total root length was much higher in the order of ca. 30,000 cm. The active root length thus only represented 0.7%–2.5% of the total root length. In reality, all roots might take up water, but at variable rates. For instance, Ahmed et al. (2018) showed that in mature maize most of the water uptake are taken up by crown roots were seminal roots and their lateral had a minor contribution to root water uptake. In addition, L might compensate experimental errors in measuring the soil conductivity or in assuming that soil and rhizosphere hydraulic properties are similar. Therefore, these values are fitting parameters and they should be cautiously interpreted.

Note also that active root length and root conductance are physically linked to each other, i.e., the longer the root, the larger its interface to soil and the bigger its conductance. These two variables were treated as independent in this study but this could be further investigated using allometric relations (Meunier et al., 2017; Meunier et al., 2018).

The relation of estimated plant hydraulic conductivity and imposed matric potential for each replication showed that the soil-plant hydraulic conductance was constant in the wet soil and that the drop in soil-plant hydraulic conductance observed at increasing transpiration rate and decreasing soil water content were well explained by the loss of soil hydraulic conductivity around the roots taking up water. Due to pressurization, xylem cavitation was likely to be prevented during the measurements and thus the decrease in conductivity was caused by soil drying.

In conclusion, we have shown that stomatal regulation reduces transpiration when soil-plant hydraulic conductance drops, preventing marked nonlinearities in the relationship between leaf water potential and transpiration rate, as hypothesized in Sperry and Love (2015). Soil-plant hydraulic conductance decreased at high transpiration rates and low soil water contents, as predicted by hydraulic models (Sperry et al., 1998). This result provides novel experimental evidence supporting the use of soil-plant hydraulic models to predict stomatal response to soil drying. Compared to studies focusing on xylem vulnerability (e.g., Anderegg et al., 2017), here we focused on soil drying as the cause of hydraulic limitation. Contrary to Anderegg et al. (2017), who found that stomata close much before the xylem cavitates, we found that stomata close when the soil hydraulic conductivity dropped. It means that for the tested maize in the silt-sand mixture, loss of soil hydraulic conductivity is the primary constraint to transpiration.

## Data Availability Statement

All datasets generated for this study are included in the article/**Supplementary Material**.

## Author Contributions

FH carried out the experiments and drafted the manuscript. MAA and AC participated in the design of the experimental setup and results evaluation. MZ and MJ helped in simulation of data. GC contributed in revision and performing statistical analysis. MZ and MJ helped in simulation of data.

## Funding

FH was funded by the Ministry of Higher Education Commission, Pakistan under contract No. 50015636. GC was funded by BMBF, project 02WIL1489 (Deutsche Israelische Wassertechnologie Kooperation). The authors acknowledge the Deutsche Forschungsgemeinschaft (DFG, German Research Foundation) for funding of the priority program 2089, project numbers 403670197 “Emerging effects of root hairs and mucilage on plant scale soil water relations” to MAA, MJ and AC.

## Conflict of Interest

The authors declare that the research was conducted in the absence of any commercial or financial relationships that could be construed as a potential conflict of interest.

## Supplementary Material

Figure S1 **(A)** Transpiration measurements using the sap flow sensor and balance; **(B)** plant in the pressure chamber with sap flow sensor connected; **(C)** water bleeding from the cut leaf.

Figure S2 Schematic of the model used for simulation of leaf water potential. Here, *ψ_b_*, *ψ_r,s_* and *ψ_leaf,x_* are the matric flux potential in the bulk soil, soil-root interface and in leaf xylem, respectively.

Figure S3 Brooks and Corey parameterization of hydraulic properties of soil: **(A)** fitted soil water retention curve, **(B)** fitted hydraulic conductivity curve. The dots show water the potential and the hydraulic conductivity of soil at different measured water contents for each replication.

Figure S4 Calibration of transpiration rates measured by sap flow with gravimetric measurements.

Figure S5 Measured xylem suction and transpiration rate for replication 2 & 3.

Table S1 The analysis of variance (N-way ANOVA) for the influence of different factors on transpiration rate (P < 0.001***, P < 0.01**, P < 0.05*).

Table S2 The analysis of variance (N-way ANOVA) for the influence of different factors on k* (P < 0.001***, P < 0.01**, P < 0.05*).

## References

[B1] Ahmed M.A. Zarebanadkouki M. Meunier F. Javaux M. Kaestner A. Carminati A. (2018). Root type matters: measurement of water uptake by seminal, crown, and lateral roots in maize. J. Exp. Bot. 69, 1199–1206. 10.1093/jxb/erx439 29304205 PMC6019006

[B2] Anderegg W. R. L. Wolf A. Arango-Velez A. Choat B. Chmura D. J. Jansen S. (2017). Plant water potential improves prediction of empirical stomatal models. PloS One 12, 1–17. 10.1371/journal.pone.0185481 PMC5638234 29023453

[B3] Bates L. M. Hall A. E. Sciences P. (1981). Stomatal closure with soil water depletion not associated with changes in bulk leaf water status. Oecologia 50, 62–65. 10.1007/BF00378794 28310062

[B4] Brodribb T. J. McAdam S. A. M. (2017). Evolution of the stomatal regulation of plant water content. Plant Physiol. 174, 639–649. 10.1104/pp.17.00078 28404725 PMC5462025

[B5] Brooks R. H. Corey A. T. (1964). Hydraulic properties of porous media (USA: Color. State Univ. Hydrol. Pap. Collins). https://doi.org/citeulike-article-id:711012

[B6] Buckley T. N. (2017). Modeling stomatal conductance. Plant Physiol. 174, 572–582. 10.1104/pp.16.01772 28062836 PMC5462010

[B7] Carminati A. Moradi A. B. Vetterlein D. Vontobel P. Lehmann E. Weller U. (2010). Dynamics of soil water content in the rhizosphere. Plant Soil 332, 163–176. 10.1007/s11104-010-0283-8

[B8] Carminati A. Vetterlein D. Koebernick N. Blaser S. Weller U. Vogel H. J. (2013). Do roots mind the gap? Plant Soil 367, 651–661. 10.1007/s11104-012-1496-9

[B9] Carminati A. Passioura J. B. Zarebanadkouki M. Ahmed M. A. Ryan P. R. Watt M. (2017). Root hairs enable high transpiration rates in drying soils. New Phytol. 216, 771–781. 10.1111/nph.14715 28758687

[B10] Carter J. N. Jensen M. E. Traveller D. J. (1980). Effect of mid- and late-season water stress on sugarbeet growth and yield. Agron. J. 72, 806–815. 10.2134/agronj1980.00021962007200050028x

[B11] Chaumont F. Tyerman S. D. (2014). Aquaporins: highly regulated channels controlling plant water relations. Plant Physiol. 164, 1600–1618. 10.1104/pp.113.233791 24449709 PMC3982727

[B12] Comstock J. P. (2002). Hydraulic and chemical signalling in the control of stomatal conductance and transpiration. J. Exp. Bot. 53, 195–200. 10.1093/jexbot/53.367.195 11807122

[B13] Cowan I. R. (1965). Transport of water in the soil-plant-atmosphere system. J. Appl. Ecol. 2, 221–239. 10.2307/2401706

[B14] Draye X. Kim Y. Lobet G. Javaux M. (2010). Model-assisted integration of physiological and environmental constraints affecting the dynamic and spatial patterns of root water uptake from soils. J. Exp. Bot. 61, 2145–2155. 10.1093/jxb/erq077 20453027

[B15] Ehlert C. Maurel C. Tardieu F. Simonneau T. (2009). Aquaporin-mediated reduction in maize root hydraulic conductivity impacts cell turgor and leaf elongation even without changing transpiration. Plant Physiol. 150, 1093–1104. 10.1104/pp.108.131458 19369594 PMC2689965

[B16] Gardner W. R. Ehlig C. F. (1963). The influence of soil water on transpiration by plants. J. Geophys. Res. 68, 5719–5724. 10.1029/JZ068i020p05719

[B17] Gardner W. R. (1960). Dynamic aspects of water availability to plants. Soil Sci. 89 (2) 63–73 10.1097/00010694-196002000-00001

[B18] Hayat F. Ahmed M. A. Zarebanadkouki M. Cai G. Carminati A. (2018). Measurements and simulation of leaf xylem water potential and root water uptake in heterogeneous soil water contents. Adv. Water Resour. 124, 96–105. 10.1016/J.ADVWATRES.2018.12.009

[B19] Knipfer T. Besse M. Verdeil J. L. Fricke W. (2011). Aquaporin-facilitated water uptake in barley (Hordeum vulgare L.) roots. J. Exp. Bot. 62, 4115–4126. 10.1093/jxb/err075 21441404 PMC3153672

[B20] Mencuccini M. Manzoni S. Christoffersen B. (2019). Modelling water fluxes in plants: from tissues to biosphere. New Phytol. 222, 1207–1222. 10.1111/nph.15681 30636295

[B21] Meunier F. Couvreur V. Draye X. Vanderborght J. Javaux M. (2017). Towards quantitative root hydraulic phenotyping: novel mathematical functions to calculate plant-scale hydraulic parameters from root system functional and structural traits. J. Math. Biol. 75, 1133–1170. 10.1007/s00285-017-1111-z 28255663 PMC5591877

[B22] Meunier F. Zarebanadkouki M. Ahmed M. A. Carminati A. Couvreur V. Javaux M. (2018). Hydraulic conductivity of soil-grown lupine and maize unbranched roots and maize root-shoot junctions. J. Plant Physiol. 227, 31–44. 10.1016/j.jplph.2017.12.019 29395124

[B23] Meyer W. S. Green G. C. (1980). Water use by wheat and plant indicators of available soil water1. Agron. J. 72, 253–257. 10.2134/agronj1980.00021962007200020002x

[B24] Passioura J. B. (1980). The transport of water from soil to shoot in wheat seedlings. J. Exp. Bot. 31, 333–345. 10.1093/jxb/31.1.333

[B25] Peters A. Durner W. (2008). Simplified evaporation method for determining soil hydraulic properties. J. Hydrol. 356, 147–162. 10.1016/j.jhydrol.2008.04.016

[B26] Pickard W. F. (1981). The ascent of sap in plants. Prog. Biophys. Mol. Biol. 37, 181–229. 10.1016/0079-6107(82)90023-2

[B27] Redondo E. Hachez C. Parent B. Tardieu F. Chaumont F. Simonneau T. (2009). Drought and abscisic acid effects on aquaporin content translate into changes in hydraulic conductivity and leaf growth rate: a trans-scale approach. Plant Physiol. 149, 2000–2012. 10.1104/pp.108.130682 19211703 PMC2663730

[B28] Schindler U. Durner W. Unold G. V. Mueller L. Wieland R. (2010). The evaporation method: extending the measurement range of soil hydraulic properties using the air-entry pressure of the ceramic cup. J. Plant Nutr. Soil Sci. 173, 563–572. 10.1002/jpln.200900201

[B29] Schröder T. Javaux M. Vanderborght J. Vereecken H. (2007). Comment on “Root water extraction and limiting soil hydraulic conditions estimated by numerical simulation”. Vadose Zone J. 6, 524–526. 10.2136/vzj2007.0042l

[B30] Simonneau T. Ehlert C. Maurel C. Tardieu F. (2009). Integrated control of leaf growth by cell turgor in response to combinations of evaporative demands and aquaporin-mediated reductions in root hydraulic conductivity. Comp. Biochem. Physiol. Part A Mol. Integr. Physiol. 153A, S228. 10.1016/j.cbpa.2009.04.572

[B31] Sinclair T. R. Holbrook N. M. Zwieniecki M. A. (2005). Daily transpiration rates of woody species on drying soil. Tree Physiol. 25, 1469–1472. 10.1093/treephys/25.111469 16105814

[B32] Sperry J. S. Love D. M. (2015). What plant hydraulics can tell us about responses to climate-change droughts. New Phytol. 207, 14–27. 10.1111/j.1469-8137.2010.03195.x 25773898

[B33] Sperry J. S. Adler F. R. Campbell G. S. Comstock J. P. (1998). Limitation of plant water use by rhizosphere and xylem conductance: results from a model. Plant Cell Environ. 21, 347–359. 10.1046/j.1365-3040.1998.00287.x

[B34] Sperry J. S. Wang Y. Wolfe B. T. Mackay D. S. Anderegg W. R. L. Mcdowell N. G. (2016). Pragmatic hydraulic theory predicts stomatal responses to climatic water deficits. New Phytol. 212, 577–589. 10.1111/nph.14059 27329266

[B35] Tardieu F. Davies W. J. (1993). Integration of hydraulic and chemical signalling in the control of stomatal conductance and water status of droughted plants. Plant Cell Environ. 16, 341–349. 10.1111/j.1365-3040.1993.tb00880.x

[B36] van Lier Q. de J. Metselaar K. van Dam J. C. (2006). Root water extraction and limiting soil hydraulic conditions estimated by numerical simulation. Vadose Zone J. 5, 1264–1277. 10.2136/vzj20060056

[B37] Van Lier Q. D. J. Van Dam J. C. Metselaar K. De Jong R. Duijnisveld W. H. M. (2008). Macroscopic root water uptake distribution using a matric flux potential approach. Vadose Zone J. 7, 1065–1078. 10.2136/vzj20070083

