# Supplementary figures and images for: Transpiration Reduction in Maize (Zea mays L) in Response to Soil Drying
Source: Front Plant Sci. 2020 Jan 23;10:1695. doi: 10.3389/fpls.2019.01695 (PMC6989490; doi:10.3389/fpls.2019.01695)

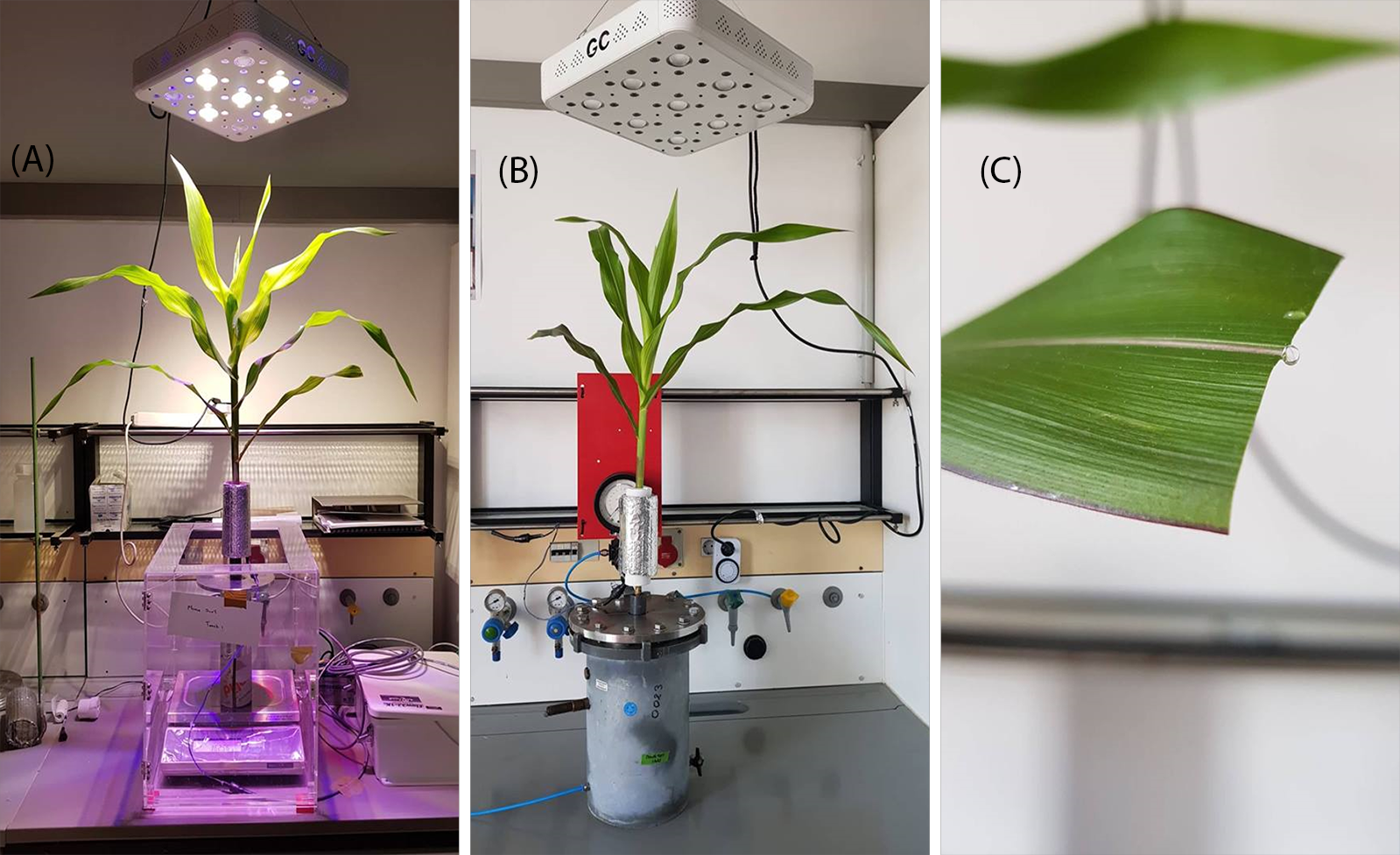

Supplement: Figure S1 — (A) Transpiration measurements using the sap flow sensor and balance; (B) plant in the pressure chamber with sap flow sensor connected; (C) water bleeding from the cut leaf. [file Image_1.tif]

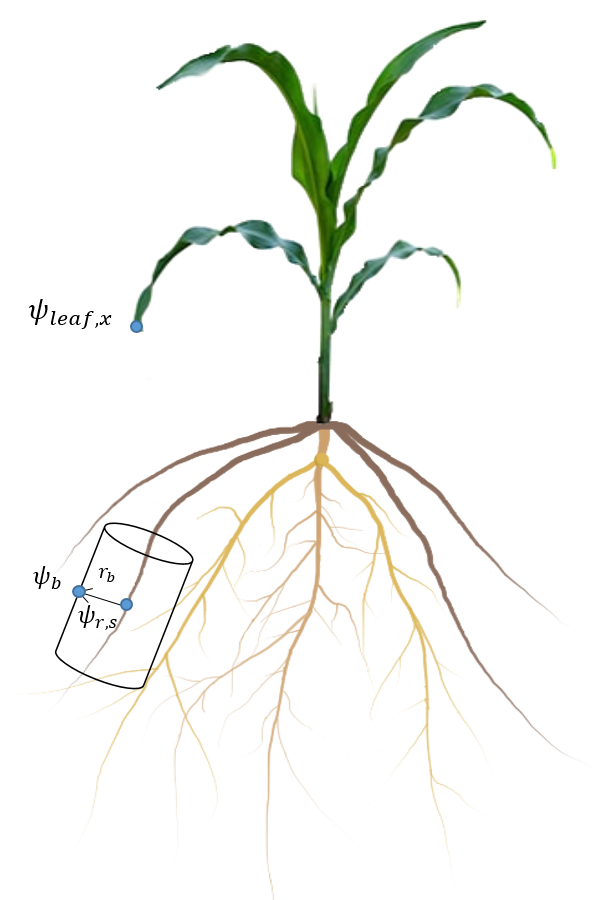

Supplement: Figure S2 — Schematic of the model used for simulation of leaf water potential. Here, ψb, ψr,s and ψleaf,x are the matric flux potential in the bulk soil, soil-root interface and in leaf xylem, respectively. [file Image_2.tif]

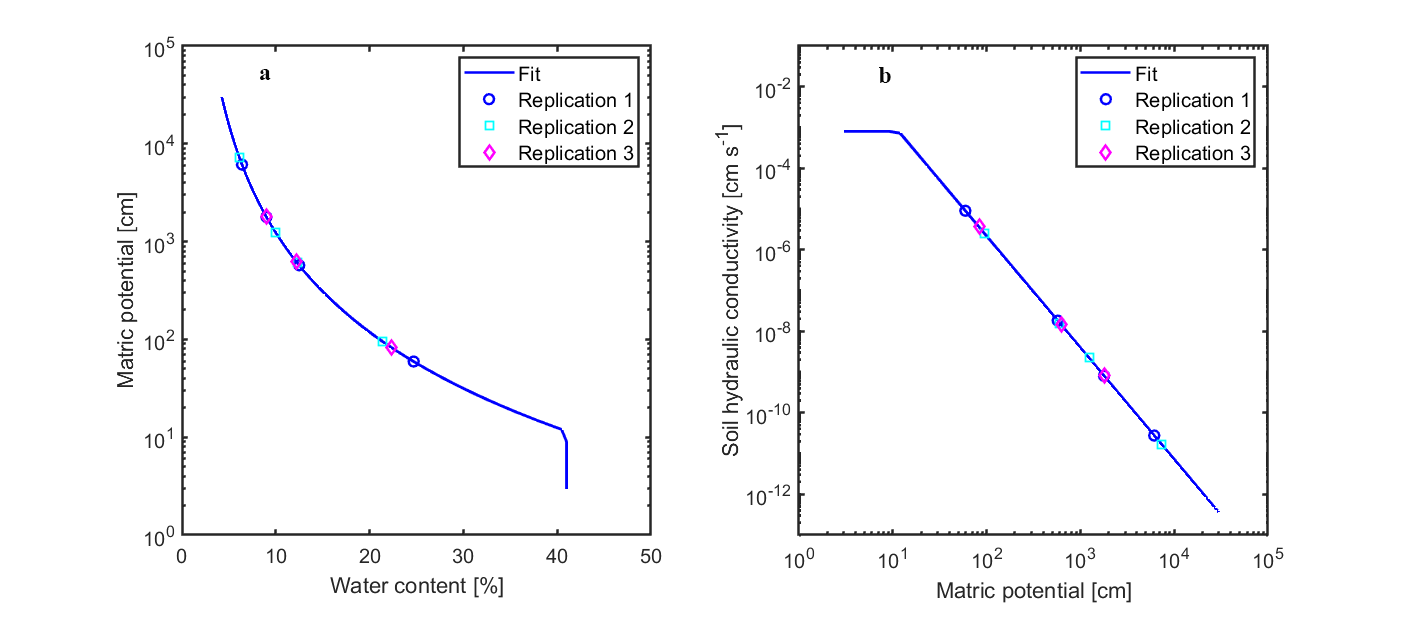

Supplement: Figure S3 — Brooks and Corey parameterization of hydraulic properties of soil: (A) fitted soil water retention curve, (B) fitted hydraulic conductivity curve. The dots show water the potential and the hydraulic conductivity of soil at different measured water contents for each replication. [file Image_3.tif]

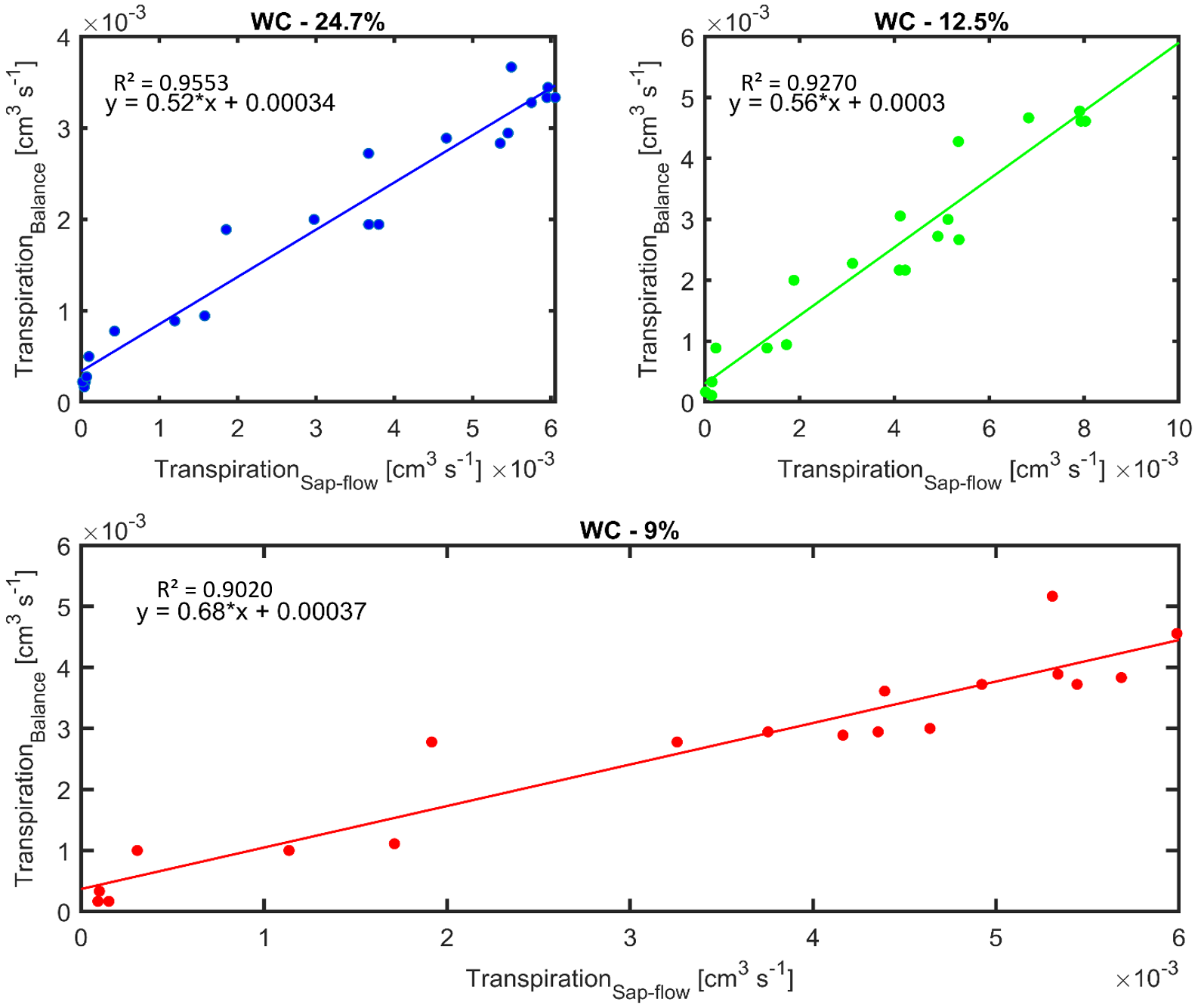

Supplement: Figure S4 — Calibration of transpiration rates measured by sap flow with gravimetric measurements. [file Image_4.tif]

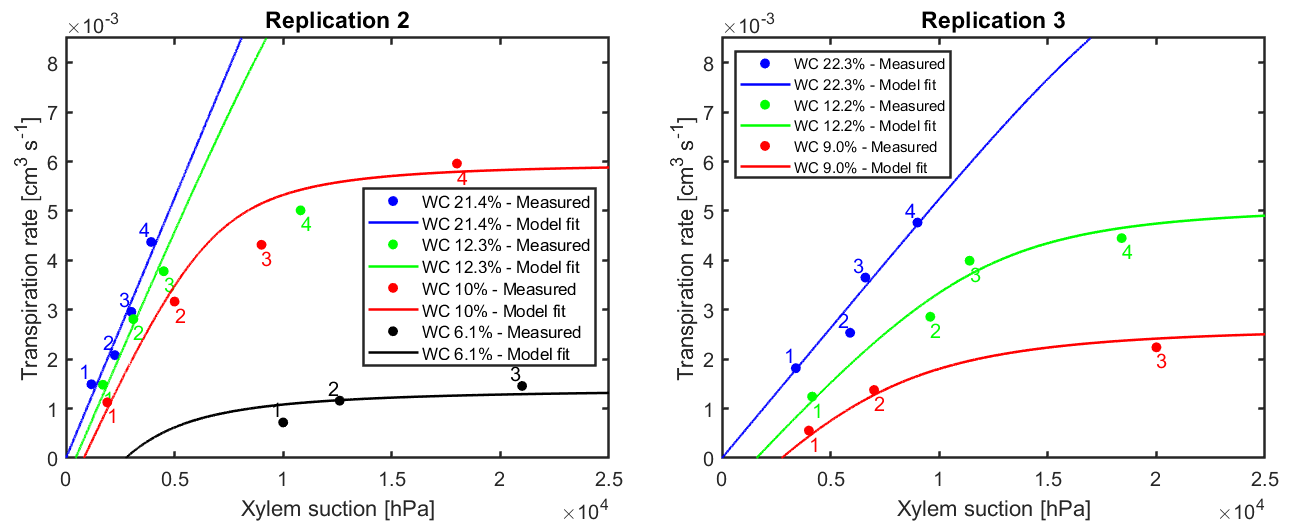

Supplement: Figure S5 — Measured xylem suction and transpiration rate for replication 2 & 3. [file Image_5.tif]

## Slide 1
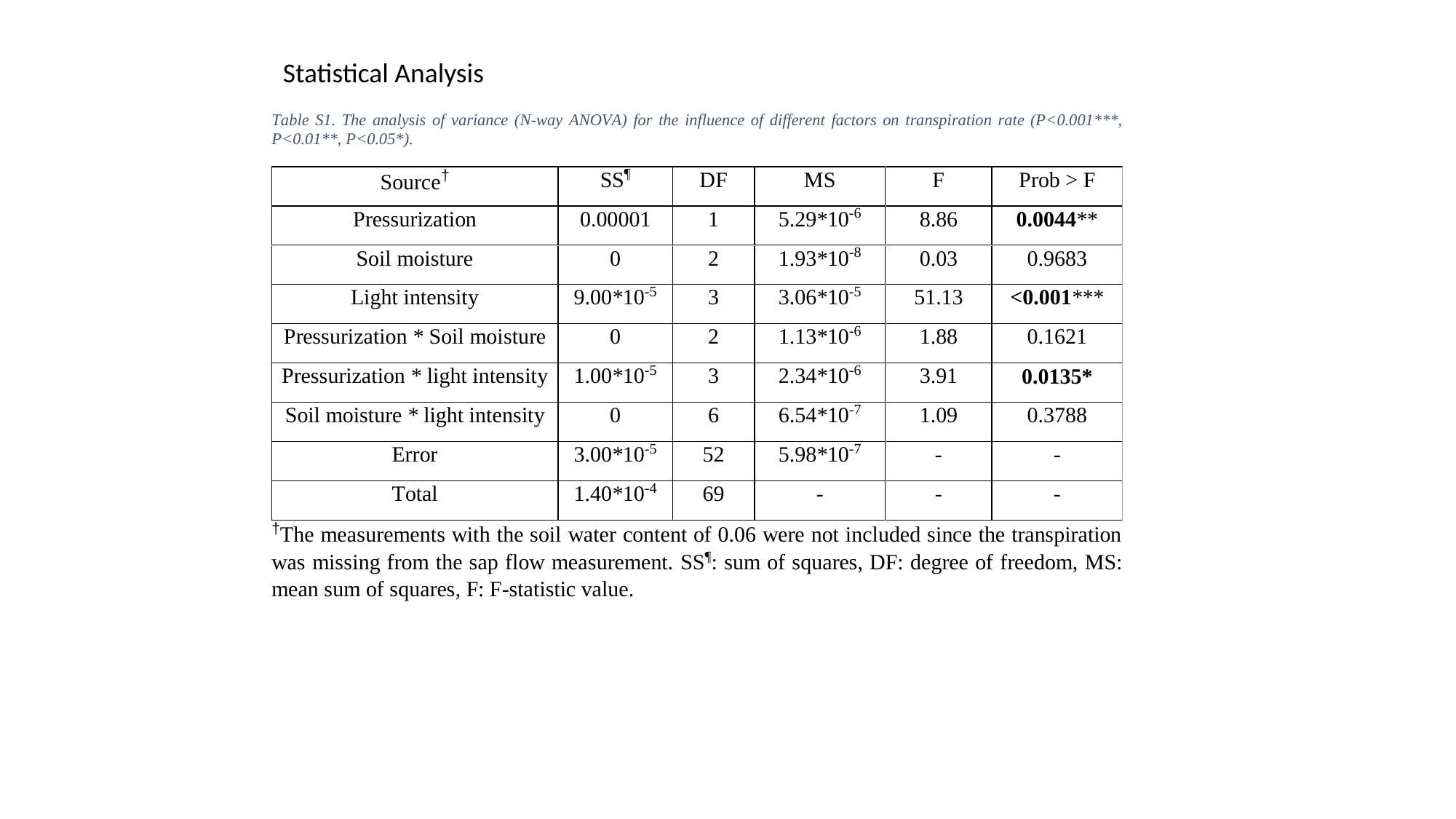

Statistical Analysis

## Slide 2
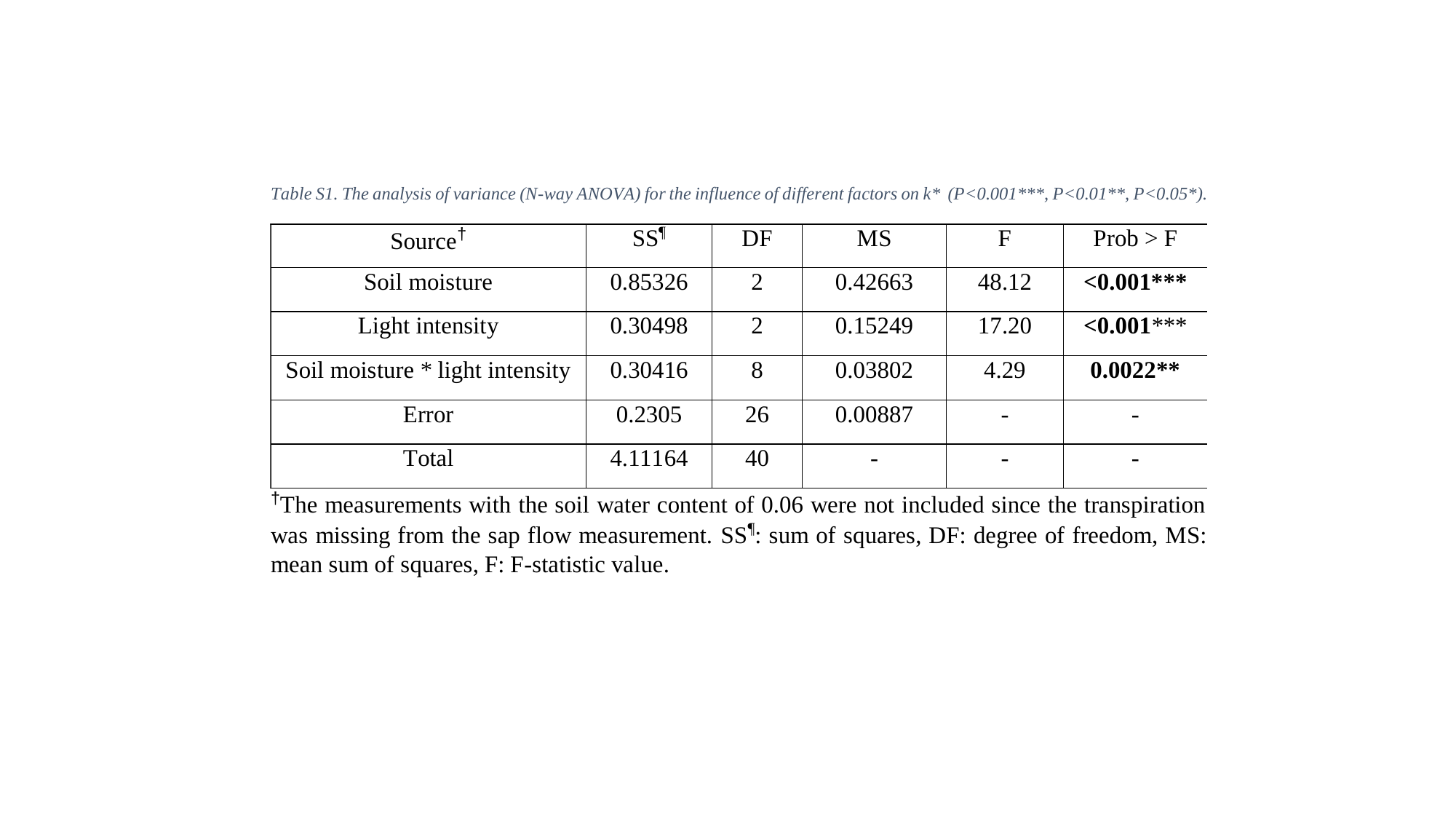

Supplement: Table S1 — The analysis of variance (N-way ANOVA) for the influence of different factors on transpiration rate (P < 0.001***, P < 0.01**, P < 0.05*). [file Presentation_1.pptx]
